# A rare case of benign pneumatosis intestinalis in Sjogren’s syndrome

**DOI:** 10.1093/jscr/rjad346

**Published:** 2023-06-17

**Authors:** Chi F Tsang, Brandon Davis, Daniel L Chan, David Yeo

**Affiliations:** Department of General Surgery, St George Hospital, Sydney, NSW, Australia; Department of General Surgery, St George Hospital, Sydney, NSW, Australia; Department of General Surgery, St George Hospital, Sydney, NSW, Australia; University of New South Wales, Sydney, NSW, Australia; Department of General Surgery, St George Hospital, Sydney, NSW, Australia

## Abstract

Pneumatosis intestinalis (PI)—the presence of intramural bowel gas—is an uncommon radiological finding, the severity of which depends on the underlying pathological process, ranging from benign disease to life-threatening ischaemia and intra-abdominal sepsis. PI has been described in systemic sclerosis and mixed connective tissue disease; however, few cases have been reported in Sjogren’s syndrome (SjS). The exact pathogenesis of PI in systemic connective tissue disorders is not fully understood and likely multifactorial. We have described a unique case of PI without evidence of peritonitis in a stable patient with long-standing SjS managed non-operatively. An awareness of such benign PI, particularly amongst patients with systemic connective tissue disease, is crucial for diagnostic accuracy and safe patient care, particularly in preventing unnecessary surgical intervention.

## INTRODUCTION

Pneumatosis intestinalis (PI)—the presence of intramural bowel gas—is an uncommon radiological finding, the severity of which depends on the underlying pathological process, ranging from benign disease to life-threatening ischaemia and intra-abdominal sepsis [1]. Benign PI has been described in obstructive lung disease, medication-related (steroids and chemotherapy), and in connective tissue disease [2]. Notably, PI has been described in systemic sclerosis and mixed connective tissue disease; however, few cases have been reported in Sjogren’s syndrome (SjS) [3].

## CASE STUDY

**Figure 1:**
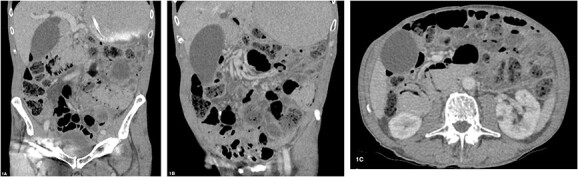
(**A**–**C**) Abdominal CT scan with oral and intravenous contrast revealing PI with gastric and proximal small bowel dilatation, without evidence of perforation or ischaemia.

An 84-year-old male with untreated primary SjS was referred to the emergency department with acute abdominal pain, distension and vomiting. He presents on the background of oesophageal dysmotility, sinus bradycardia and previous appendicectomy. On this presentation, he was clinically well, haemodynamically stable and afebrile. His abdomen was distended but otherwise soft and benign to examination. Laboratory findings were as follows: white cell count 5 × 10^9^/L, haemoglobin 130 g/L, C-reactive protein level 6 mg/L and lactate 1.0 mmol/L. Dual contrast abdominal CT scan (Fig. 1) demonstrated marked distension of stomach, duodenum and proximal small bowel without obvious transition point. Pneumatosis was evident from the second part of duodenum to proximal small bowel without evidence of bowel perforation. After evaluation and discussion of the discordant radiological and clinical findings, the patient was treated for small bowel obstruction with nasogastric tube decompression and placed nil by mouth. He responded well to conservative management and was discharged home after 3 days with resolution of obstruction. Serial imaging 2 months later demonstrated almost complete resolution of pneumatosis.

## DISCUSSION

The exact pathogenesis of PI in systemic connective tissue disorders is not fully understood and likely multifactorial. Of note, bacterial overgrowth and increased intra-luminal hydrogen may compromise mucosal integrity and facilitate the intramural translocation of gas, with rupture of resulting sub-serosal gas blebs by causing an associated benign pneumoperitoneum [4]. The mechanical theory suggests that increased intraluminal pressure forces gas into the submucosal space via mucosal breaches [5]. Alternatively, vasculitis-related micro-ischaemia and long-term corticosteroid administration may induce atrophy of intestinal mucosa and allow gas translocation in SLE [5].

Irrespective of the pathophysiology, the clinical importance of benign PI is the risk of misdiagnosis leading to unnecessary operative intervention [6]. Discernible identifiers for pathological PI include hypotension, peritonitis and lactataemia; however, CT features may be more useful in subtle presentations and comprise portal venous gas, ascites and poor bowel enhancement [7, 8]. For benign PI, treatment is conservative in most cases, and it is crucial to consider other diagnoses, such as small bowel obstruction in the present case, as a cause of the patients’ clinical presentation. Treatment options for benign PI include inspired oxygen therapy, oral metronidazole and elemental diet; fewer than 5% of patients require surgery compared with its emergent role in pathological PI [9, 10].

Here, we have described a unique case of PI without evidence of peritonitis in a stable patient with long-standing SjS managed non-operatively. An awareness of such benign PI, particularly amongst patients with systemic connective tissue disease, is crucial for diagnostic accuracy and safe patient care, particularly in preventing unnecessary surgical intervention.
